# Polydopamine-Based “Four-in-One” Versatile Nanoplatforms for Targeted Dual Chemo and Photothermal Synergistic Cancer Therapy

**DOI:** 10.3390/pharmaceutics11100507

**Published:** 2019-10-01

**Authors:** Gan Liu, Nansha Gao, Yun Zhou, Junpeng Nie, Wei Cheng, Miaomiao Luo, Lin Mei, Xiaowei Zeng, Wenbin Deng

**Affiliations:** 1 School of Pharmaceutical Sciences (Shenzhen), Sun Yat-sen University, Shenzhen 518107, China; liugan5@mail.sysu.edu.cn (G.L.); gaonsh@mail.sysu.edu.cn (N.G.); zhouy599@mail2.sysu.edu.cn (Y.Z.); upc201412@163.com (W.C.); luomm3@mail2.sysu.edu.cn (M.L.); meilin7@mail.sysu.edu.cn (L.M.); 2 Division of Life and Health Sciences, Graduate School at Shenzhen, Tsinghua University, Shenzhen 518055, China; niejunpeng@126.com

**Keywords:** synergistic cancer therapy, polydopamine, aptamer, dual drug delivery, photothermal therapy

## Abstract

The development of versatile nanoscale drug delivery systems that integrate with multiple therapeutic agents or methods and improve the efficacy of cancer therapy is urgently required. To satisfy this demand, polydopamine (PDA)-modified polymeric nanoplatforms were constructed for the dual loading of chemotherapeutic drugs. The hydrophobic anticancer drug docetaxel (DTX) was loaded into the polymeric nanoparticles (NPs) which were fabricated from the star-shaped copolymer CA-PLGA. Then DTX-loaded NPs were coated with PDA, followed by conjugation of polyelethyl glycol (PEG)-modified targeting ligand aptamer AS1411(Apt) and adsorption of the hydrophilic anticancer drug doxorubicin (DOX). This “four-in-one” nanoplatform, referred to as DTX/NPs@PDA/DOX-PEG-Apt, demonstrated high near-infrared photothermal conversion efficiency and exhibited pH and thermo-responsive drug release behavior. Furthermore, it was able to specifically target MCF-7 human breast carcinoma cells and provide synergistic chemo-photothermal therapy to further improve the anticancer effect both in vitro and in vivo, providing a novel promising strategy for cancer therapy.

## 1. Introduction

In the past few years, although many treatments [1,2,3,4], such as chemotherapy, radiotherapy, gene therapy, phototherapy and immunotherapy, have been widely investigated, cancers cannot be cured by any monotherapy regimens in many cases. This can mainly be attributed to tumor heterogeneity and mutability [5] that weaken the overall anticancer efficacy or induce drug resistance [6,7]. The antitumor effects may be further improved via combining more than two therapies with different mechanisms [8,9,10,11,12]. Nevertheless, direct administration of two or more drugs suffers from not only the low delivery efficiency of small molecular drugs, but also the great difficulty of co-delivery into one cell. Recently, nanoscale drug delivery systems (10–200 nm) have been verified to augment the anticancer activities of drugs, as they enrich in solid tumors through the enhanced permeability and retention (EPR) effect [13,14,15,16,17,18,19]. Various nanocarriers, including liposomes, polymeric nanoparticles and mesoporous silica nanoparticles, have been reported to load two or more drugs as well as to combine different therapies (e.g., photothermal therapy). Liposomes can load different kinds of drugs in both hydrophobic bilayer and hydrophilic cavity [20,21,22], but they suffer from low stability and uncontrollable drug release. Although polymeric nanocarriers can effectively load drugs in their hydrophobic cores [23,24], co-loading of different kinds of drugs is rather difficult. Mesoporous silica nanoparticles have been widely employed to load various drugs through considerable pores [25,26], but they are hardly applicable due to non-biodegradability. Therefore, exploring novel versatile nanoplatforms integrating different therapeutic agents or methods is of great significance.

Polydopamine (PDA), a melanin-like mimic of mussel adhesive protein formed by the oxidation of dopamine, has attracted considerable attention in the biomedical field because of its unique advantages, including excellent biocompatibility and biodegradability, and its spontaneous deposition on the surfaces of biomaterials to form a PDA film [27,28,29,30]. In particular, PDA has many advantages in applications of nanoscale drug delivery systems. Firstly, PDA films have a dense crosslinked structure that can entirely encapsulate drug nanocarriers, increasing their in vivo stability and avoid premature drug release [31,32,33]. Secondly, the large number of quinone groups on the surface of PDA is able to react easily with amino- and thiol-containing materials, enabling surface modification such as conjugation with PEG and tumor-targeting ligands [31,34,35,36]. Thirdly, a PDA surface could efficiently adsorb drugs, in particular DOX, through π–π stacking and achieve highly pH-responsive controlled release [37,38]. Lastly, PDA possesses high near-infrared (NIR) photothermal conversion efficiency, which suggests that it is a potentially efficacious agent for photothermal therapy [32,39,40]. Thus PDA-coated nanocarriers could achieve tumor-targeted dual drug delivery and synergistic chemo-photothermal therapy, which has not yet been reported for cancer therapy.

**Figure 1:**
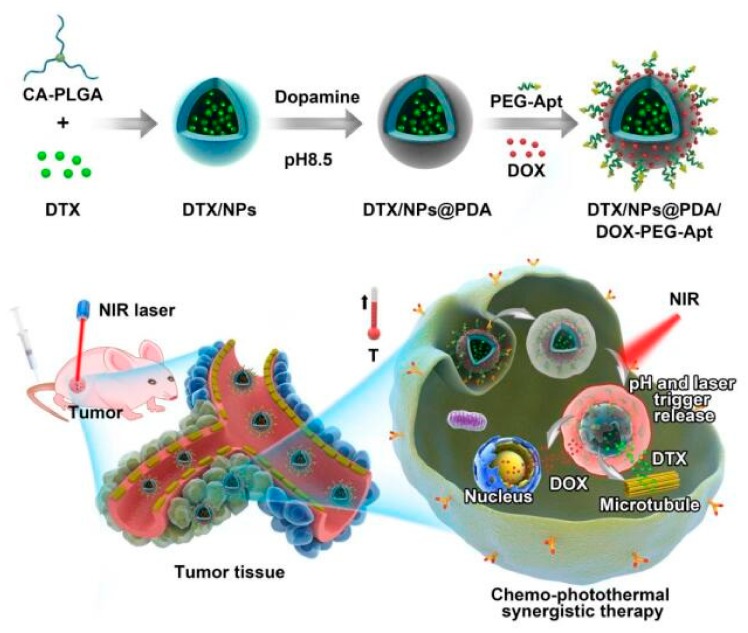
Schematic illustration of docetaxel (DTX)/polymeric nanoparticles (NPs)@polydopamine (PDA)/doxorubicin (DOX)-polyelethyl glycol (PEG)-Aptamer (Apt) for targeted dual chemo and photothermal synergistic cancer therapy.

Herein, we report on the performance of a PDA-coated “four-in-one” polymeric nanoplatform for targeted dual drug delivery and synergistic chemo and photothermal therapy of breast cancer. As shown in Figure 1, the biodegradable star-shaped polymer CA-PLGA was used as a polymeric carrier to load the hydrophobic anticancer drug docetaxel (DTX) as it has a higher drug loading efficiency than linear PLGA [41]. After surface coating with PDA, DTX-loaded nanoparticles (DTX/NPs) were coupled with the polyelethyl glycol (PEG)-modified targeting ligand aptamer AS1411 (Apt) [32,35]. PDA-coated NPs can adsorb the anticancer drug DOX, thereby enabling the simultaneous loading of DTX (which acts on microtubules) and DOX (which acts on nuclei). After their intravenous injection, targeting NPs accumulate in tumor sites, become ingested by tumor cells and then provide chemo–photothermal therapy under NIR irradiation.

## 2. Materials and Methods

### 2.1. Materials

The star-shaped polymer CA-PLGA and the following nanoparticles were synthesized according to previously work [41]. DTX was obtained from Shanghai Jinhe Bio-Technology Co., Ltd. (purity: 99.9%, Shanghai, China). Dopamine hydrochloride, dimethyl sulfoxide (DMSO), 2-(4-Amidinophenyl)-6-indolecarbamidine dihydrochloride (DAPI) and 3-(4,5-dimethylthiazol-2-yl)-2,5-diphenyltetrazolium bromide (MTT) agent were bought from Sigma-Aldrich (St. Louis, MO, USA). PEG-NH_2_, FITC-PEG-NH_2_ and Mal-PEG-NH_2_ were obtained from Shanghai Yare Biotech, Inc. (Shanghai, China). AS1411 (Apt-SH: 5′-GGT GGT GGT GGT TGT GGT GGT GGT GG-3′-thiol) was obtained from GenePharma Co., Ltd. (Suzhou, Jiangsu, China). Doxorubicin hydrochloride was obtained from Dalian Meilun Biology Technology Co., Ltd. (Dalian, China). The human breast carcinoma cell line MCF-7 was purchased from the American Type Culture Collection (ATCC, Rockville, MD).

### 2.2. Formulation of DTX-Loaded NPs

NPs loading with DTX (DTX/NPs) were prepared by nanoprecipitation in acetone/water mixed solvent system according to our previous work [41]. Briefly, after dissolving CA-PLGA (100 mg) and DTX (10 mg) in acetone (8 mL), the mixed solution was dripped slowly to 0.03% (*w/v*) TPGS aqueous solution (100 mL) under stirring at room temperature, and the mixture was further stirred overnight to evaporate the acetone. Through centrifuging the mixture at 20,000 rpm for 15 min, the precipitate was washed triplicate with DI water to obtain DTX/NPs, followed by lyophilization for further use.

### 2.3. Surface Coating with PDA

Surface-coating with PDA on DTX/NPs (DTX/NPs@PDA) was achieved under weak alkaline conditions (pH 8.5). 1 mg/mL DTX/NPs were suspended in Tris buffer (10 mM, pH 8.5). Dopamine hydrochloride was then added under stirring at room temperature (0.5 mg/mL). The color of the mixture gradually turned black after stirring for 5 h. After centrifugation at 20,000 rpm for 15 min, the precipitate was washed three times with deionized water to obtain DTX/NPs@PDA NPs, followed by lyophilization for further use.

### 2.4. Surface Conjugation of Ligands to PDA-coated NPs

The targeting ligand, NH_2_-PEG-Apt, was synthesized by reacting Mal-PEG-NH_2_ with AS1411 (Apt-SH) via a Michael addition reaction. The targeting ligand was conjugated under weak alkaline conditions (pH 8.5) to obtain DTX/NPs@PDA-PEG-Apt. Briefly, DTX/NPs@PDA (1 mg/mL) was suspended in Tris buffer (10 mM, pH 8.5). Then Apt-PEG-NH_2_ (2 mg/mL) was added into the solution followed by stirring for a further 5 h. After centrifugation at 20,000 rpm for 15 min, the precipitate was washed in triplicate with DI water to obtain DTX/NPs@PDA-PEG-Apt NPs, followed by lyophilization for further use.

### 2.5. Adsorption of DOX onto the PDA Layer

Briefly, 50 mg DTX/NPs@PDA-PEG-Apt was resuspended in deionized water (1 mg/mL). 10 mL DOX aqueous solution (5 mg/mL) was then added under stirring and the mixture was further stirred overnight to completely absorb the DOX. After centrifugation at 20,000 rpm for 15 min, the precipitate was washed three times with DI water to obtain DTX/NPs@PDA/DOX-PEG-Apt, followed by lyophilization for further use.

### 2.6. Characterization of NPs

Dynamic light scattering (DLS) was used to evaluate the size distribution and zeta potential of the NPs (Zetasizer Nano ZS90, Malvern Instruments Ltd., Worcestershire, UK). Transmission electron microscopy (TEM) images were obtained using FEI Tecnai G2 F30 transmission electron microscope. X-ray photoelectron spectroscopy (XPS) data were collected using Al Kα radiation (hν = 1486.58 eV) by Kratos Axis Ultra DLD spectrometer. Fourier transform infrared (FT-IR) spectra were carried out by the Thermo Nicolet spectrometer (Madison, Wisconsin), using KBr to create a pellet. To quantify the drug loading capacity (LC) of NPs, the supernatant of each step was collected to measure the concentration of drugs by HPLC (LC 1200, Agilent Technologies, Santa Clara, CA, USA). A reverse-phase C-18 column (150 × 4.6 mm, 5 μm, C18, Agilent Technologies, Santa Clara, CA, USA) was used with the flow rate of the mobile phase as 1.0 mL/min. For DTX, the mobile phaseconsisted of acetonitrile and DI water (50:50). For DOX, the mobile phase consisted of phosphate buffer, methanol and acetonitrile (30:20:50). UV-Vis detector was used to detect DTX or DOX at 227 nm and 233 nm, respectively. The concentration of drug was calculated according to the standard curve of the drug and LC (%) was calculated using the following equation:

$$\mathrm{LC}\left(\%\right)=\frac{\mathrm{Weight}\;\mathrm{of}\;\mathrm{DTX}/\mathrm{DOX}\;\mathrm{in}\;\mathrm{NPs}}{\mathrm{Weight}\;\mathrm{of}\;\mathrm{NPs}}\;\times \;100\%.$$

### 2.7. Evaluation of Photothermal Effect

An 808 nm NIR laser was used to irradiate different NPs (DTX/NPs, DTX/NPs@PDA and DTX/NPs@PDA/DOX-PEG-Apt) for 5 min. The temperature of the samples were measured by an IR thermal image camera every 15 s to quantify the photothermal conversion efficiency of the NPs. Temperature curves were plotted, using DI water as the reference. DTX/NPs@PDA/DOX-PEG-Apt was also irradiated in varying concentrations (2.5, 25, 125, 250 and 500 μg/mL) at 1.5 W/cm^2^ and in varying laser power (0.5, 1, 1.5 and 2 W/cm^2^) at 250 μg/mL. Next, the photothermal stability of the NPs was tested by irradiating for 5 min and four cycles at 250 μg/mL and 1.5 W/cm^2^ followed by cooling to room temperature.

### 2.8. In Vitro Drug Release Study

The dialysis method was utilized to monitor the in vitro drug release of NPs. Briefly, drug-loaded NPs (5 mg) were dispersed into PBS containing 0.1% Tween-80 (1 mL). Then the mixture was added in a dialysis bag (MWCO = 3500, Shanghai Sangon, China) and then immersed in 15 mL PBS at different pH (pH 7.4 or 5.0) in a 50 mL centrifuge tube. Further, the centrifuge tube was placed in a 37 °C water bath under shaking. At specified time intervals, 1 mL of dialyzate was fetched from the centrifuge tube and an equal volume of PBS was replenished. Then 1 mL of diethyl ether was added to dialyzate to extract the drug. After removing the aqueous phase, the organic phase was evaporated by introducing N_2_. Then 1 mL of mobile phase was added to dissolve the drug and 20 μL of the solution was used for HPLC. The drug concentrations were calculated from standard curves and an in vitro release curve was plotted. The drug release profiles were also performed before and after laser irradiation at 1.5 W/cm^2^ for 5 min at specified time intervals.

### 2.9. Cellular Uptake of Fluorescent NPs

FITC-labeled NPs were synthesized by adding H_2_N-PEG-FITC along with H_2_N-PEG-Apt. After treated with FITC-NPs for 0.5 h or 2 h, MCF-7 cells were washed three times with PBS, fixed with 4% paraformaldehyde for 15 min and then stained with DAPI for 10 min. Confocal laser scanning microscopy (CLSM, Olympus Fluoview FV-1000, Tokyo, Japan) was utilized to observe the cellular uptake of NPs. The excitation wavelength of blue, green and red channels was 340, 485 and 488 nm, respectively.

In addition, the cellular uptake was quantified by the flow cytometry (FCM). MCF-7 cells (1 × 10^6^ cells/well) were seeded into six-well plates and incubated overnight. Then fluorescent NPs (250 μg/mL) were added into the plates and co-cultured with the cells for 1 h. After that, the cells were carefully digested, washed with PBS and resuspended in PBS for FACS. The excitation and emission wavelength was 488 nm and 530 nm, respectively. The fluorescence of approximately 10,000 cells was measured for each analysis.

### 2.10. Cell Viability Study

The cytotoxicity of NPs towards MCF-7 cells was measured by MTT assay. After seeded into a 96-well plate, the MCF-7 cells (1 × 10^4^ cells/well) were cultured overnight. Then drug-loaded NPs with DTX concentration of 0.25, 2.5, 12.5 and 25 μg/mL and drug-free or DOX-loaded NPs with the same NP concentration were added and cultured for another 24 h and 48 h. MTT solution (20 μL, 5 mg/mL) was then added to each well followed by culturing the cells for further 4 h. The cell viability was evaluated by MTT at each time point. The survival rate of each group of cells was calculated by reference to a cell control group with a survival rate of 100%. The IC_50_ values are defined as the drug concentration causing the death of 50% of a cell population after a specified period of time.

The combination index (CI) is used to evaluate the synergistic effect of combination therapies. According to the reference [42], we calculated the combination index (CI) with respect to experimental parameters (IC_50_) by using the formula CI = C_DTX,50_/IC_50,DTX_ + C_DOX,50_/IC_50,DOX_. C_DTX,50_ and C_DOX,50_ refers to the drug concentration of DTX and DOX when the combined drug group caused the 50% cell inhibition. IC_50,DTX_ and IC_50,DOX_ means the drug concentration causing 50% inhibition with single DTX or DOX. If CI < 1, it suggests that different drugs have a synergistical effect; otherwise, that is, if CI = 1 or CI > 1, the drugs have additive or antagonistical effect.

### 2.11. Cellular Transport Mechanism Study

Immunofluorescence experiments were used to study colocalization of the early endosomal marker EEA1 with fluorescent NPs. MCF-7 cells (2 × 10^4^ cells/well) were seeded in a glass bottom cell culture dish and cultured overnight. Then the cells were treated with fluorescent NPs for 2 h, fixed with 4% paraformaldehyde and blocked with 3% BSA. The cells were incubated with a primary antibody against EEA1, successively with a secondary rhodamine-labeled antibody (TRITC) and observed by CLSM. Then cells were transfected with a DeRed-Rab7 plasmid and then treated with fluorescent NPs for 2 h. Cells were fixed in 4% paraformaldehyde and finally visualized by CLSM. After the transfection or treatment with fluorescent NPs, the cells were treated with Lyso-Tracker Red for 1 h to detect the lysosomes, washed three times with PBS, fixed with 4% paraformaldehyde and observed by CLSM.

### 2.12. Pharmacokinetic Analysis

All animal experimental protocols were given permission by the Administrative Committee on Animal Research in Sun Yat-sen University. In vivo experiments were all complied with the guidelines of the institutional animal ethics committee. The project identification code is SYSU-IACUC-2018-B3215 and the date is December 23rd 2018. Pharmacokinetic analysis was performed on 5–6 week old male SD rats (230 ± 10 g). The rats were randomly divided into equal groups. Drug-loaded NPs were injected through the tail vein at a DTX dose of 10 mg/kg. Blood was collected in 200 μL aliquots by eyelid sampling at regular time intervals and then centrifuged at 4000 rpm for 10 min to obtain plasma. The DTX in the plasma was extracted using 1 mL of diethyl ether, which was fully evaporated followed by adding 1 mL of HPLC flow phase. By reference to a standard curve of DTX, its concentration in plasma was plotted against time. Similarly, the pharmacokinetics of DOX was also tested.

### 2.13. Animals and Tumor Model Establishment

From the Laboratory Animal Center of Sun Yat-sen University, 5–6 weeks old female sever combined immunodeficient (SCID) mice were purchased. After feeding in an specific pathogen free (SPF) class experimental animal room for 1–2 weeks, each mouse (18–20 g) was injected with 100 μL of MCF-7 cells (2 × 10^6^ cells) subcutaneously into the back to establish the MCF-7 cells xenograft model. Vernier caliper was used to measure the volume of the tumor (V) followed by calculation by equaton: V = A × B^2^/2, where A and B respectively refers to the length and width of the tumor.

### 2.14. In Vivo Imaging

To assess the effects of photothermal therapy, an infrared (IR) thermal image camera was performed to monitor the nude mice. The drug-loaded NPs were injected via tail vein injection at a dose of 1 mg/kg. After 24 h, the tumor site was irradiated with 808 nm laser at 1.5 W/cm^2^ for 5 min during which time the mouse was imaged every 15 s and its tumor temperature was recorded.

### 2.15. In Vivo Antitumor Efficacy Study

The mice were randomly divided into 6 groups (*n* = 5) when the tumor volume grew to 50 mm^3^. Over 14 days of treatment, 100 μL of saline and other formulation NPs were injected via tail vein every four days (10 mg/kg body weight on a DTX basis) and NIR laser irradiation was carried out to the NIR treatment group after 24 h of injection. The volume of tumors was recorded by calipers and their weight was measured every two days. After the mice were sacrificed on 14 day, their tumor tissues were separated and weighed. Then the major organs (heart, lung, liver, spleen and kidney) and tumors were all collected followed by fixing in 10% neutral formalin for histological analysis. Then after embedding in paraffin, the tissues were sliced into approximately 4 μm sections, stained with H&E and then analyzed by light microscopy.

### 2.16. Statistical Methodology

SPSS 22.0 software was used for data analysis. The experimental data was expressed as x ± s. T-tests were used to compare between two groups. The test significance level (α) was 0.05. *p < 0.05, **p < 0.01, ***p < 0.001.

## 3. Results and Discussion

### 3.1. Preparation and Characterization of NPs

The size and zeta potential of NPs are important parameters affecting their stability and the EPR effect, respectively. Studies have shown that NPs with a diameter in the range 10–200 nm are most likely to become taken up by tumor tissue through EPR effects [2]. The results of DLS were shown in Table 1 and Figure S1 (cf. Supplementary Data). The diameter of the drug-loaded NPs was 100–200 nm with a small polydispersity index (PDI), indicating that their size distribution was relatively uniform. As shown in Figure S2, the zeta potential of the NPs was negative, which would be favorable for prolonging circulation before enrichment in tumor tissue [43,44]. Furthermore, the drug loading content of all single-loaded NPs was approximately 10%. After optimization, the loading contents of two types of drugs in DTX/NPs@PDA/DOX-PEG-Apt were both 8–9%, allowing optimal synergistic effect of dual drugs.

**Figure 2:**
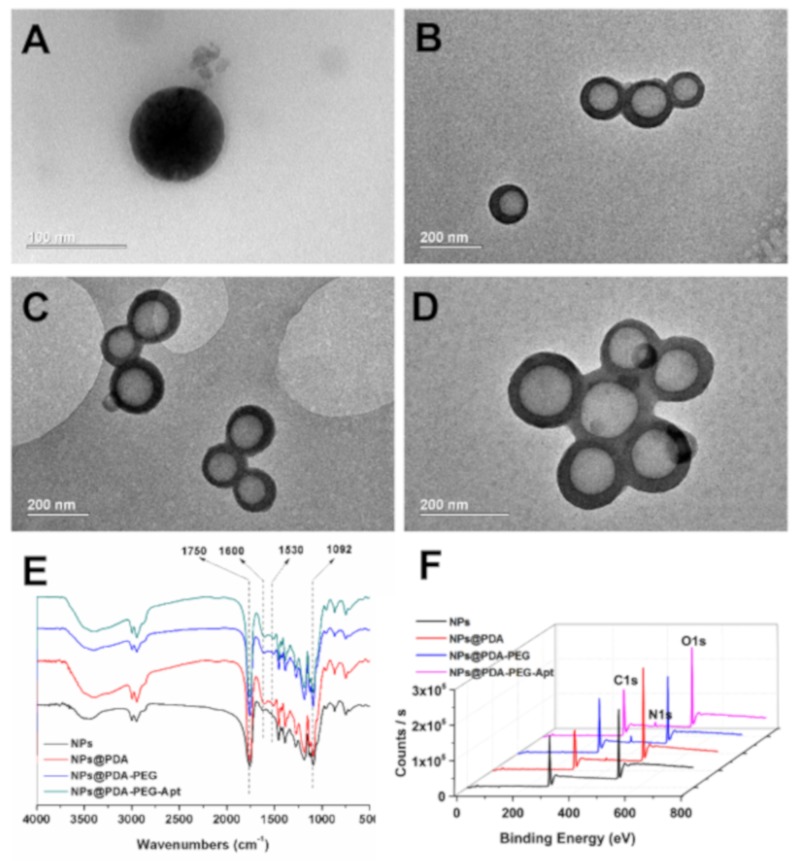
TEM images of (**A**) DTX/NPs, (**B**) DTX/NPs@PDA, (**C**) DTX/NPs@PDA-PEG-Apt and (**D**) DTX/NPs@PDA/DOX-PEG-Apt. (**E**) Fourier transform infrared (FT-IR) spectra of drug-free NPs. (**F**) X-ray photoelectron spectroscopy (XPS) wide scan spectra of drug-free NPs.

The morphology and surface properties of NPs were characterized by TEM, FT-IR spectra and XPS. As shown in Figure 2A–D, all NPs were smooth nanospheres, in which the PDA-coated NPs obviously possessed core-shell structures. TEM showed the thickness of PDA layer is around 30 nm (Figure 2B), similar to our previous work [40]. The FT-IR spectra displayed the following characteristics (Figure 2E): (1) strong peaks could be found at 1750 cm^−1^ in FT-IR spectra of all samples, representing the carbonyl group in CA-PLGA; (2) after surface coating, the double peaks at 1600 cm^−1^ and 1530 cm^−1^ can be attributed to C=C resonance and N–H bending vibrations in the aromatic ring, indicating that the surface of the NPs had been coated with PDA [36]; (3) after surface coating, the broad peak between 3600 cm^−1^ and 3300 cm^−1^ was assigned to a N–H/O–H stretching vibration, indicating that the amino-containing ligand was attached to the surface of the PDA-coated NPs. Furthermore, XPS results exhibited that a pronounced nitrogen peak appeared after coating with PDA (Figure 2F), while this nitrogen peak was significantly enlarged after functionalization with amino-containing ligand (Figure S3). These results all indicate that the NPs were successfully coated with PDA and conjugated with the targeting ligand.

### 3.2. Photothermal Effect and Drug Release Profiles of NPs

**Figure 3:**
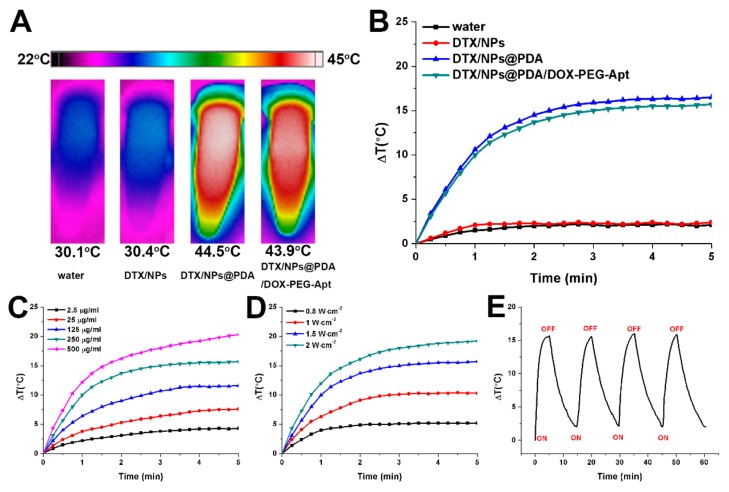
(**A**) IR thermal image of water, DTX/NPs, DTX/NPs@PDA, and DTX/NPs@PDA/DOX-PEG-Apt solution under continuous NIR laser irradiation (808 nm, 1.5 W/cm^2^) for 5 min. (**B**) Temperature curves of water, DTX/NPs, DTX/NPs@PDA, and DTX/NPs@PDA/DOX-PEG-Apt solution under continuous NIR laser irradiation (808 nm, 1.5 W/cm^2^) for 5 min. (**C**) Temperature curves of the DTX/NPs@PDA/DOX-PEG-Apt solution with different concentrations under continuous NIR laser irradiation (808 nm, 1.5 W/cm^2^) for 5 min. (**D**) Temperature curves of the DTX/NPs@PDA/DOX-PEG-Apt solution (250 μg/mL) under various power intensities. (**E**) Temperature curve of the DTX/NPs@PDA/DOX-PEG-Apt solution (250 μg/mL) under four NIR laser on/off cycles (808 nm, 1.5 W/cm^2^).

The photothermal conversion efficiency of the NPs was evaluated with varying laser powers and NP concentrations. As shown in Figure 3A,B, after NIR laser irradiation (1.5 W/cm^2^) for 5 min, there was no apparent temperature increase in the aqueous environment or DTX/NPs, while the temperature of the PDA-coated NPs increased significantly. These results indicate that the drug-loaded NPs (DTX/NPs) have no photothermal property but do after PDA coating. In addition, further modification of targeted ligand and loading of DOX caused only a minimal impairment of the photothermal effect of PDA. As shown in Figure 3C,D, the photothermal effect of PDA-coated NPs depended on NP concentration and laser power. To avoid excessive laser power and unnecessary damage to normal tissue in subsequent animal experiments, NIR laser at 1.5 W/cm^2^ was chosen. As shown in Figure 3E, after four cycles of “irradiation-free cooling”, the maximum temperature and temperature curve basically maintained, indicating the robust stability of photothermal capability of the NPs after multiple irradiations.

**Figure 4:**
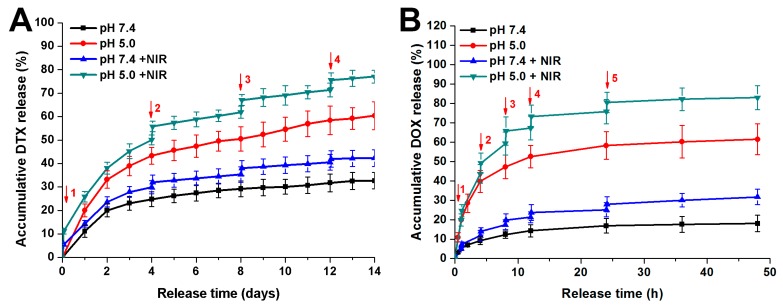
In vitro drug release profiles of DTX/NPs@PDA/DOX-PEG-Apt at different pH with or without NIR laser irradiation (808 nm, 1.5 W/cm^2^). (**A**) Accumulative DTX release. (**B**) Accumulative DOX release. ↓: NIR irradiation for 5 min.

Then the release of DTX and DOX from the NPs was tested under different pH and NIR light. Figure 4A showed that the DTX release profile of DTX/NPs@PDA/DOX-PEG-Apt exhibited obvious pH-responsiveness. Over 14 days, the ultimate drug release level was approximately 60% and 30% at pH 5.0 and pH 7.4, respectively. This would be attributed to the partially decomposition of PDA in acidic conditions, which favored to DTX release. After laser irradiation at 1.5 W/cm^2^ for 5 min every four days, the release of DTX increased by approximately 10% to 15%, exhibiting burst release after each laser irradiation. The release of DOX also exhibited remarkable pH and NIR responsive release over 48 h (Figure 4B). This pH- and NIR-responsiveness of drugs are likely to reduce the premature release during circulation as well as increase the specific release in the acidic tumor microenvironment.

### 3.3. Cellular Uptake of Fluorescent NPs

**Figure 5:**
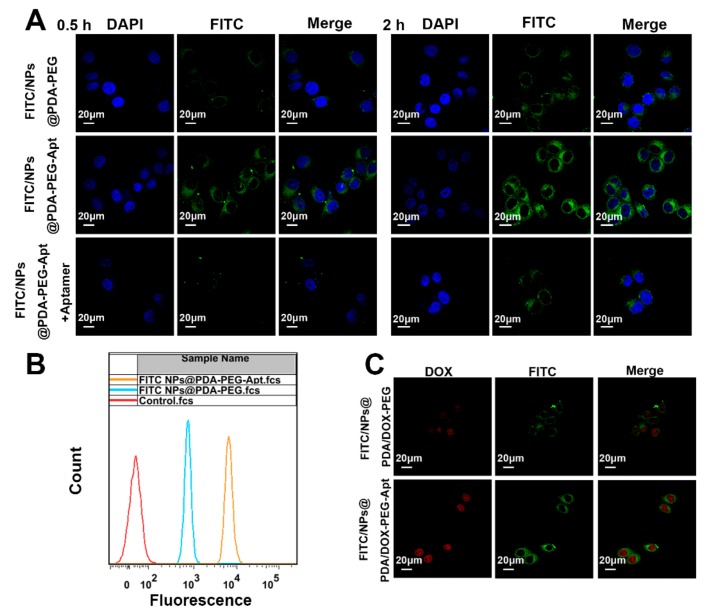
(**A**) Confocal laser scanning microscopy (CLSM) images of MCF-7 cells after incubation with FITC-labeled NPs for 0.5 and 2 h. Green: FITC-labeled NPs, blue: DAPI-stained nucleus, Scale bar: 20 μm. (**B**) Flow cytometry (FCM) analysis of MCF-7 cells after incubation with FITC-labeled NPs for 1 h. (**C**) CLSM images of MCF-7 cells after incubation with FITC/NPs@PDA/DOX-PEG or FITC/NPs@PDA/DOX-PEG-Apt for 2 h. Green: FITC-labeled NPs, Red: DOX, scale bar = 20 μm.

To study the uptake of drug-loaded NPs by tumor cells, NPs were labeled with FITC and analyzed by CLSM and FACS. MCF-7 cells were treated with FITC/NPs@PDA-PEG and FITC/NPs@PDA-PEG-Apt for 0.5 h and 2 h, respectively. Figure 5A showed that almost no green fluorescence was observed in the non-aptamer group after treatment for 0.5 h, indicating that FITC/NPs@PDA-PEG could not be uptaken by the cancer cells. Conversely, green fluorescence around the blue fluorescence of the nuclei could be clearly observed in the aptamer group, indicating that large number of FITC/NPs@PDA-PEG-Apt was uptaken into the cytoplasm within 0.5 h. After treatment for 2 h, more active targeting NPs were uptaken by cells than 0.5 h as expected. To verify whether the cellular uptake mechanism of the active targeting group was mediated by the aptamer, a control group in which the active targeting NPs and free aptamer were both added into cell cultures. As shown in Figure 5A, green fluorescence was significantly attenuated after the addition of aptamer, indicating that the uptake of active targeting NPs was associated with an aptamer-mediated endocytosis. FACS was used to further quantify the uptake efficiency. As shown in Figure 5B, after 1 h treatment with fluorescent NPs, the uptake efficiency of active targeting NPs performed much superior than that of non-targeting NPs.

As DOX exhibits red fluorescence, the uptake of DOX-loaded fluorescent NPs was observed in both green and red channels using CLSM. As shown in Figure 5C, after incubation with DOX-loaded fluorescent NPs for 2 h, the intensity of both green and red fluorescence of active targeting group were obviously higher than those of non-targeting group. These results demonstrate that the active targeting still promoted the uptake of NPs by cancer cells after surface loading of the second chemo drug.

### 3.4. Effect of NPs on Cell Viability

**Figure 6:**
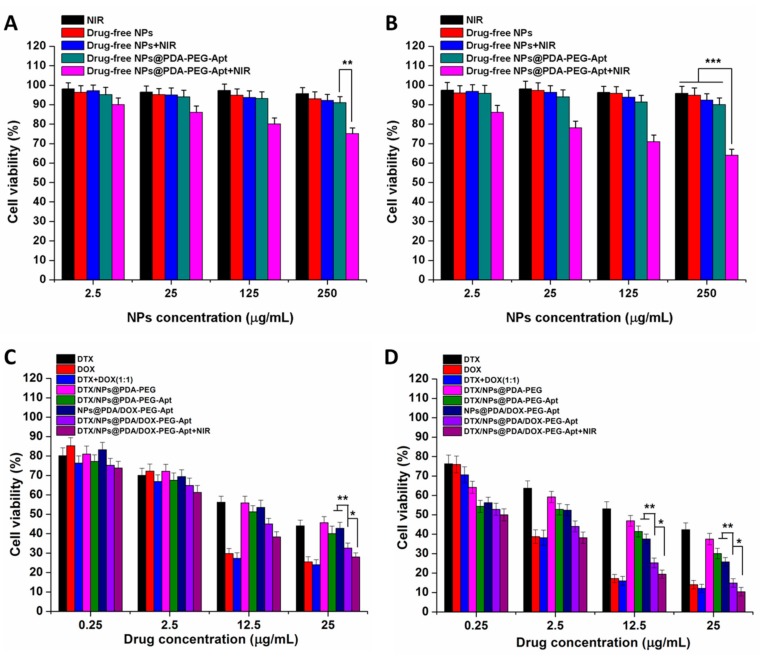
In vitro cytotoxicity on MCF-7 cells of NIR, drug-free NPs, drug-free NPs + NIR, drug-free NPs@PDA-PEG-Apt and drug-free NPs@PDA-PEG-Apt+NIR for (**A**) 24 h and (**B**) 48 h. Viability of MCF-7 cells treated with different therapies for (**C**) 24 h and (**D**) 48 h.

To evaluate the in vitro cytotoxicity of DTX/NPs@PDA/DOX-PEG-Apt, MTT assay was carried out. Firstly, the cytotoxicity of drug-free NPs and single NIR laser irradiation on cells were studied. MCF-7 cells were treated with NIR, drug-free NPs, drug-free NPs + NIR, drug-free NPs@PDA-PEG-Apt and drug-free NPs@PDA-PEG-Apt + NIR for 24 and 48 h. As shown in Figure 6A,B, the cell viability of the first four groups was higher than 95%, indicating that all of the drug-free NPs showed no cytotoxicity on MCF-7 cells. The cell viability of the fifth group was approximately 75% (24 h) and 65% (48 h), demonstrating its photothermal cytotoxicity under NIR irradiation. The cytotoxicity of drug-loaded NPs was further studied. Eight groups, including DTX, DOX, DTX + DOX (1:1), DTX/NPs@PDA-PEG, DTX/NPs@PDA-PEG-Apt, NPs@PDA/DOX-PEG-Apt, DTX/NPs@PDA/DOX-PEG-Apt and DTX/NPs@PDA/DOX-PEG-Apt + NIR were studied. As shown in Figure 6C,D: (1) all eight groups exhibited significant cytotoxicity towards MCF-7 cells, which was positively correlated with drug concentration and treatment time; (2) the combination therapy effectively improved the cancer cell killing effect; (3) the order of cell cytotoxicity of the four NP groups was: DTX/NPs@PDA/DOX-PEG-Apt+NIR > DTX/NPs@PDA/DOX-PEG-Apt > DTX/NPs@PDA-PEG-Apt ~ NPs@PDA/DOX-PEG-Apt > DTX/NPs@PDA-PEG, indicating that the active targeting, dual chemo therapy and combination with photothermal therapy greatly increased the killing effect to cancer cells.

The IC_50_ values, calculated from the MTT assay, were shown in Table 2 and Table S1. After 48 h of culture, the combination index (CI) of free DTX and DOX (1:1) was 0.84, while the CI of drugs loaded in NPs was 0.9. Both of them are less than 1. So it is confirmed that the two drugs in NPs have synergistic effects.

### 3.5. Cellular Uptake Mechanism under NIR Irradiation

**Figure 7:**
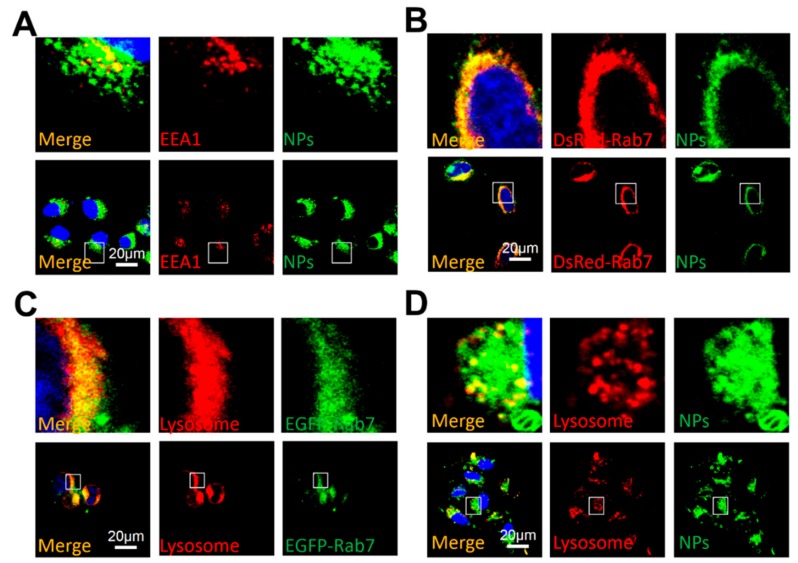
CLSM images of (**A**) MCF-7 cells treated with FITC-labeled NPs (1 mg/mL) for 20 h, in which EEA1 was tested with primary antibody against EEA1, (**B**) DsRed-Rab7 transfected MCF-7 cells treated with FITC-labeled NPs (1 mg/mL) for 20 h, (**C**) EGFP-Rab7 transfected MCF-7 cells treated with Lyso-Tracker Red probes for 1 h, (**D**) MCF-7 cells treated with FITC-labeled NPs (1 mg/mL) for 20 h followed by treated with Lyso-Tracker Red probes for 1 h. (**A**–**D**) MCF-7 cells were irradiated by NIR (808 nm, 1.5 W/cm^2^, 5 min) at 0.5 h post-treatment. Scale bar: 20 μm, NPs: FITC/NPs@PDA-PEG-Apt.

Since the aptamer AS1411 was designed to specifically bind with the nucleolin which is overexpressed on the surface of tumor cells, the AS1411-conjugated NPs were engineered to be uptaken by the tumor cells through the aptamer-mediated endocytosis. In a classical endocytosis pathway [45], NPs enter the cell sequentially through early endosomes, late endosomes and lysosomes. Since EEA1 and Rab7 are widely used as early and late endosome markers, respectively, we studied their co-localization with the fluorescent NPs FITC/NPs@PDA-PEG-Apt. As shown in Figure 7A,B, FITC/NPs@PDA-PEG-Apt did co-localize with EEA1 and Rab7, demonstrating that it was uptaken through the aptamer-mediated endocytosis and transported to both early and late endosomes. Then LysoTracker Red was used to label the late endosomes and lysosomes to study its co-localization with fluorescent NPs. As shown in Figure 7C,D, fluorescent NPs did co-localize with late endosomes and lysosomes, indicating that the NPs were delivered from late endosomes to lysosomes. The above results all demonstrated that the versatile NPs passed through early endosomes, late endosomes and lysosomes in turn through receptor-mediated endocytosis under NIR irradiation.

### 3.6. In Vivo Pharmacokinetics and Tumor Targeting

The in vivo pharmacokinetics of the chemotherapeutic drugs and drug-loaded NPs was studied. As shown in Figure S4, the plasma level of free DTX decreased rapidly after injection, whereas that of DTX in the NPs decreased much slower, particularly in the PEG-modified NPs. On the other hand, the pharmacokinetic profiles of DOX were displayed in Figure S5, also demonstrating that DTX/NPs@PDA/DOX-PEG-Apt prolonged the circulation time obviously compared to free DOX. These results all indicate that NPs would greatly prolong the half-life of the loaded dual drugs, anticipating their effective enriching in tumors through EPR effect.

**Figure 8:**
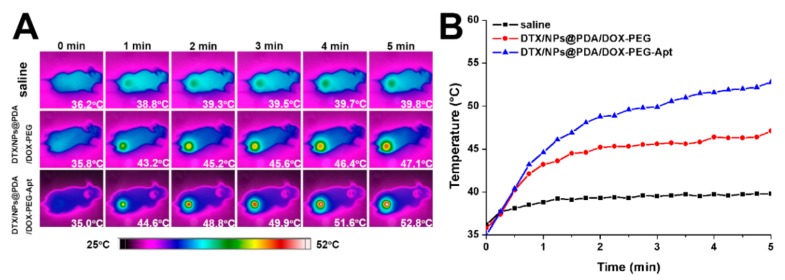
(**A**) IR thermal images of nude mice bearing MCF-7 cells xenograft under photothermal therapy. (**B**) Time-dependent temperature curves of MCF-7 tumor-bearing mice. NIR laser: 808 nm, 1.5 W/cm^2^, 5 min.

Next, to assess the tumor targeting and photothermal effect, an IR thermal imager was used to monitor the nude mice. After injection with saline, DTX/NPs@PDA/DOX-PEG and DTX/NPs@PDA/DOX-PEG-Apt through the tail vein for 24 h, the tumor sites were irradiated with NIR laser light at 1.5 W/cm^2^ for 5 min and the temperature was recorded every 15 s by thermography. The temperature of the tumor site of saline group increased slightly, whereas that of two NP groups increased by 11.3 and 17.8 °C, respectively (Figure 8), demonstrating their excellent tumor targeting and photothermal effects in vivo. Furthermore, the temperature increase of the DTX/NPs@PDA/DOX-PEG-Apt was higher than that of the DTX/NPs@PDA/DOX-PEG, confirming the specific targeting capability of the conjugated aptamer.

### 3.7. In Vivo Multimodal Antitumor Efficacy

**Figure 9:**
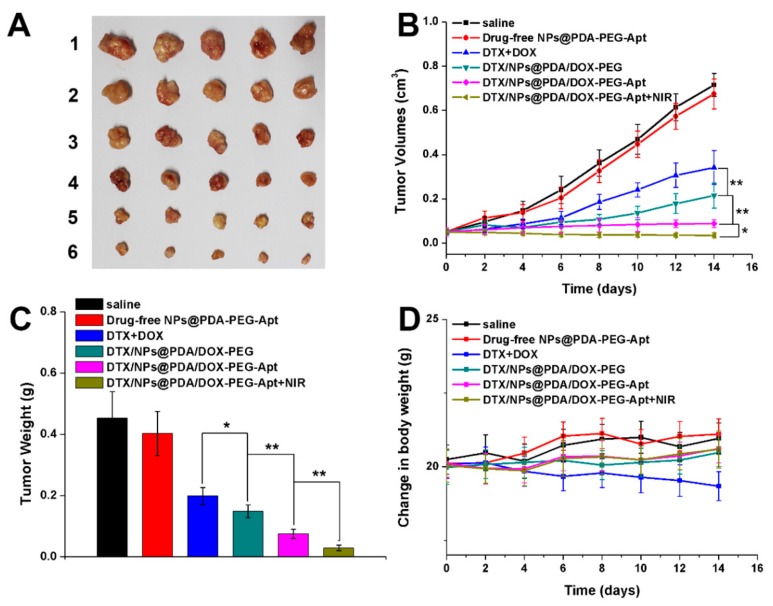
In vivo antitumor effect of different therapies on the MCF-7 cells xenograft-bearing mice (*n* = 5). (**A**) Morphology of tumors of the sacrificed mice after treatment. The numbers 1–6 refer to saline, drug-free NPs@PDA-PEG-Apt, DTX+DOX, DTX/NPs@PDA/DOX-PEG, DTX/NPs@PDA/DOX-PEG-Apt, and DTX/NPs@PDA/DOX-PEG-Apt + NIR, respectivley. (**B**) Tumor growth curves during treatment cycle. (**C**) Tumors weight of each group after treatment. (**D**) Changes of body weight during treatment cycle.

Based on the in vitro and in vivo results above, the multimodal antitumor efficacy of the NPs in vivo was evaluated. Saline, drug-free NPs@PDA-PEG-Apt, DTX + DOX, DTX/NPs@PDA/DOX-PEG, DTX/NPs@PDA/DOX-PEG-Apt and DTX/NPs@PDA/DOX-PEG-Apt + NIR were injected into mice via tail vein. Over 14 days, drug-loaded NPs were injected every four days and the NIR treatment group was irradiated with NIR laser at 24 h after injection. The tumor volume was measured by a vernier caliper and the mice were weighed every two days. After 14 days, the mice were sacrificed followed by collecting and weighting their tumors. Figure 9 showed that the tumor volumes of the saline and drug-free NPs group were much larger than that of other groups, indicating that the drug-free NPs could not inhibit the tumor growth. On the other hand, the tumor volume of the DTX + DOX group was also larger than all of the drug-loaded NP groups, indicating that the tumor inhibition effect of the drugs alone was relatively limited. In addition, the tumor growth of the DTX/NPs@PDA/DOX-PEG-Apt group was almost completely inhibited, resulting in much smaller tumor volume than that of the DTX/NPs@PDA/DOX-PEG group, demonstrating that the conjugated aptamer greatly improved the tumor inhibition of the NPs. After NIR irradiation, the tumor volume of the DTX/NPs@PDA/DOX-PEG-Apt group even decreased remarkably, indicating the excellent antitumor efficacy of dual chemo and photothermal synergistic therapy. Lastly, in contrast to the decreased body weight of the mice in the DTX + DOX group, that of the NP groups remained unchanged, indicating the good biosafety of the NPs.

Finally, H&E was used to evaluate the effect of synergistic therapy on the major organs (heart, lung, liver, spleen and kidney) and tumor tissues. Figure S6 showed no noticeable damage to the major organs in the drug-free NPs@PDA-PEG-Apt group, further confirming the biocompatibility of the NPs in mice. Whereas in the DTX/NPs@PDA/DOX-PEG-Apt + NIR group, the tumor tissue was severely destroyed, leading to much more apoptosis or necrosis than all of other groups. Therefore, the targeted dual drug delivery system, integrating with chemo and photothermal therapy, greatly suppressed tumor growth without apparent side effects, holding great potential for cancer therapy.

## 4. Conclusions

In this study, a targeted dual drug delivery system, DTX/NPs@PDA/DOX-PEG-Apt, was developed to deliver two clinical chemotherapeutic drugs, DTX and DOX. The PDA coating of the NPs surface allowed effective NIR photothermal capability and pH and NIR-responsive dual drug release to achieve multimodal synergistic antitumor effects both in vitro and in vivo, providing a novel promising strategy for cancer therapy.

## Figures and Tables

**Table 1:** Characterization of drug-loaded polymeric nanoparticles (NPs).

Samples (*n* = 3)	Size (d.nm)	PDI	ZP (mV)	DTX LC (%)	DOX LC (%)
**DTX/NPs**	105.3 ± 2.3	0.128	−21.5 ± 4.7	9.88 ± 0.41	N.A.
**DTX/NPs@PDA**	161.5 ± 3.6	0.151	−17.3 ± 5.1	9.67 ± 0.39	N.A.
**DTX/NPs@PDA-PEG**	166.3 ± 4.1	0.139	−13.6 ± 3.8	9.43 ± 0.43	N.A.
**DTX/NPs@PDA-PEG-Apt**	167.1 ± 3.9	0.145	−13.4 ± 4.1	9.39 ± 0.32	N.A.
**DTX/NPs@PDA/DOX-PEG-Apt**	170.3 ± 4.6	0.160	−10.6 ± 2.9	8.47 ± 0.27	8.61 ± 0.34

PDI: polydispersity index; ZP: zeta potential; LC: loading content; N.A.: not applicable.

**Table 2:** IC_50_ values on MCF-7 cells after incubation with DTX and DTX-loaded NPs for 24 and 48 h.

Incubation Time (h)			IC_50_ (μg/mL)			
	DTX	DTX/NPs@ PDA-PEG	DTX/NPs@ PDA-PEG-Apt	NPs@PDA/DOX-PEG-Apt	DTX/NPs@PDA/DOX-PEG-Apt	DTX/NPs@PDA/DOX-PEG-Apt+NIR
**24**	19.62	22.03	11.71	15.40	6.24	3.90
**48**	13.29	5.33	1.23	1.19	0.55	0.38

## Supplementary Materials

The following are available online at https://www.mdpi.com/1999-4923/11/10/507/s1, Figure S1: Size distribution of all NPs, Figure S2: Zeta potential of all NPs, Figure S3: XPS narrow scan spectra of N1s peaks of drug-free NPs, Figure S4: Time-dependent DTX concentration profile after intravenous administration of DTX, DTX/NPs@PDA, DTX/NPs@PDA-PEG, DTX/NPs@PDA-PEG-Apt and DTX/NPs@PDA/DOX-PEG-Apt in vivo. Figure S5: Time-dependent DOX concentration profile after intravenous administration of DOX and DTX/NPs@PDA/DOX-PEG-Apt, Figure S6: Representative H&E stained images of major organs and tumors after treated with (1) saline, (2) Drug-free NPs@PDA-PEG-Apt, (3) DTX+DOX, (4) DTX/NPs@PDA/DOX-PEG, (5) DTX/NPs@PDA/DOX-PEG-Apt and (6) DTX/NPs@PDA/DOX-PEG-Apt+NIR over 14 days, Table S1: IC50 values of DOX and DTX+DOX (1:1) on MCF-7 cells after 24 and 48 h incubation.
