# Supplementary material for: Polydopamine-Based “Four-in-One” Versatile Nanoplatforms for Targeted Dual Chemo and Photothermal Synergistic Cancer Therapy
Source: Pharmaceutics. 2019 Oct 1;11(10):507. doi: 10.3390/pharmaceutics11100507 (PMC6835447; doi:10.3390/pharmaceutics11100507)
Supplement: Supplementary file 1 [file pharmaceutics-11-00507-s001.pdf]

pharmaceutics

MDPI

Supplementary Materials: Polydopamine-Based "Four-in-One" Versatile Nanoplatforms for Targeted Dual Chemo and Photothermal Synergistic Cancer Therapy

Gan Liu, Nansha Gao, Yun Zhou, Junpeng Nie, Wei Cheng, Miaomiao Luo, Lin Mei, Xiaowei Zeng and Wenbin Deng

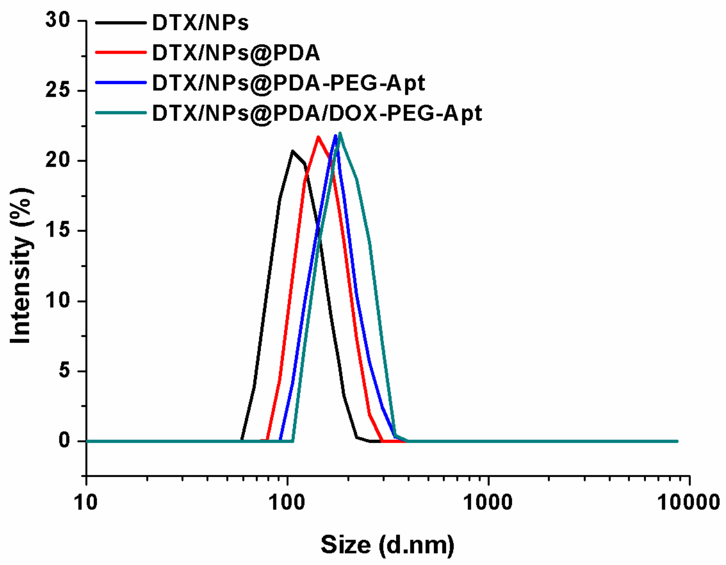

Figure S1. Size distribution of all NPs.

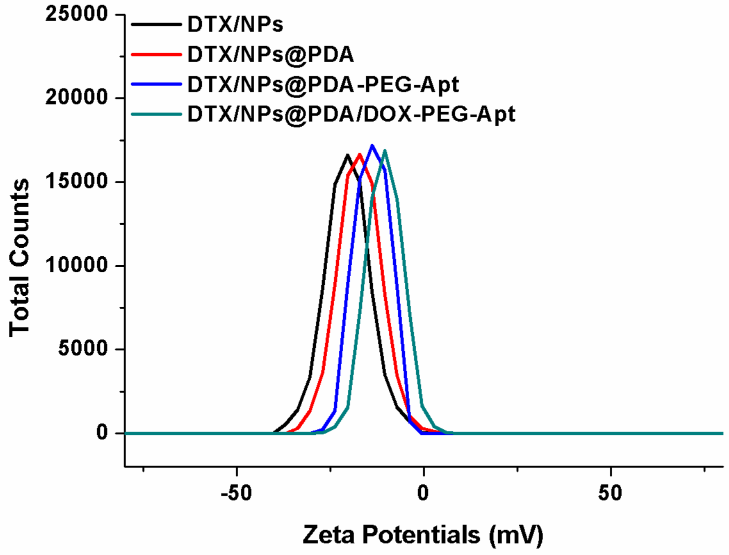

Figure S2. Zeta potential of all NPs.

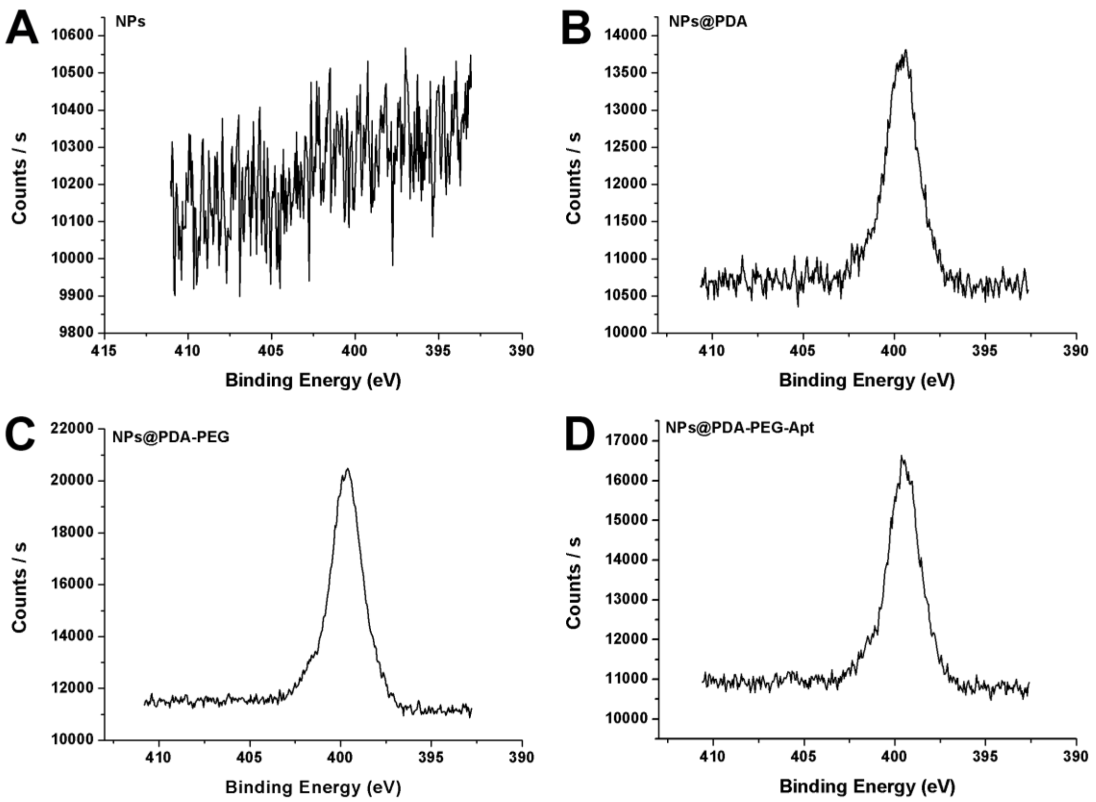

Figure S3. XPS narrow scan spectra of N1s peaks of drug-free NPs.

Table S1. IC_{50} values of DOX and DTX+DOX (1:1) on MCF-7 cells after 24 and 48 h incubation.

Incubation Time (h)	IC_{50} (μg/ml)	
	DOX	DTX+DOX (1:1)
**24**	5.00	3.32
**48**	1.35	1.03

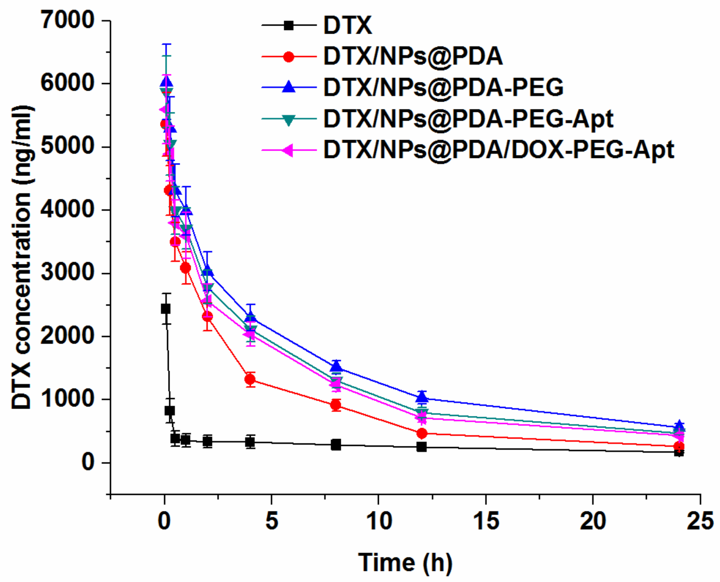

Figure S4. Time-dependent DTX concentration profile after intravenous administration of DTX, DTX/NPs@PDA, DTX/NPs@PDA-PEG, DTX/NPs@PDA-PEG-Apt and DTX/NPs@PDA/DOX-PEG-Apt in vivo.

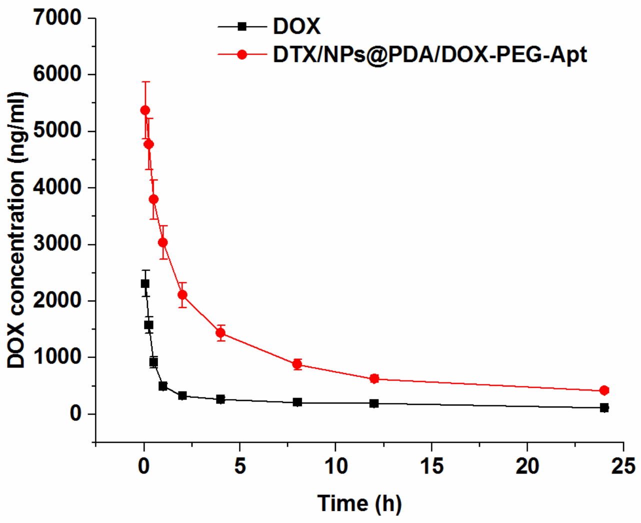

Figure S5. Time-dependent DOX concentration profile after intravenous administration of DOX and DTX/NPs@PDA/DOX-PEG-Apt.

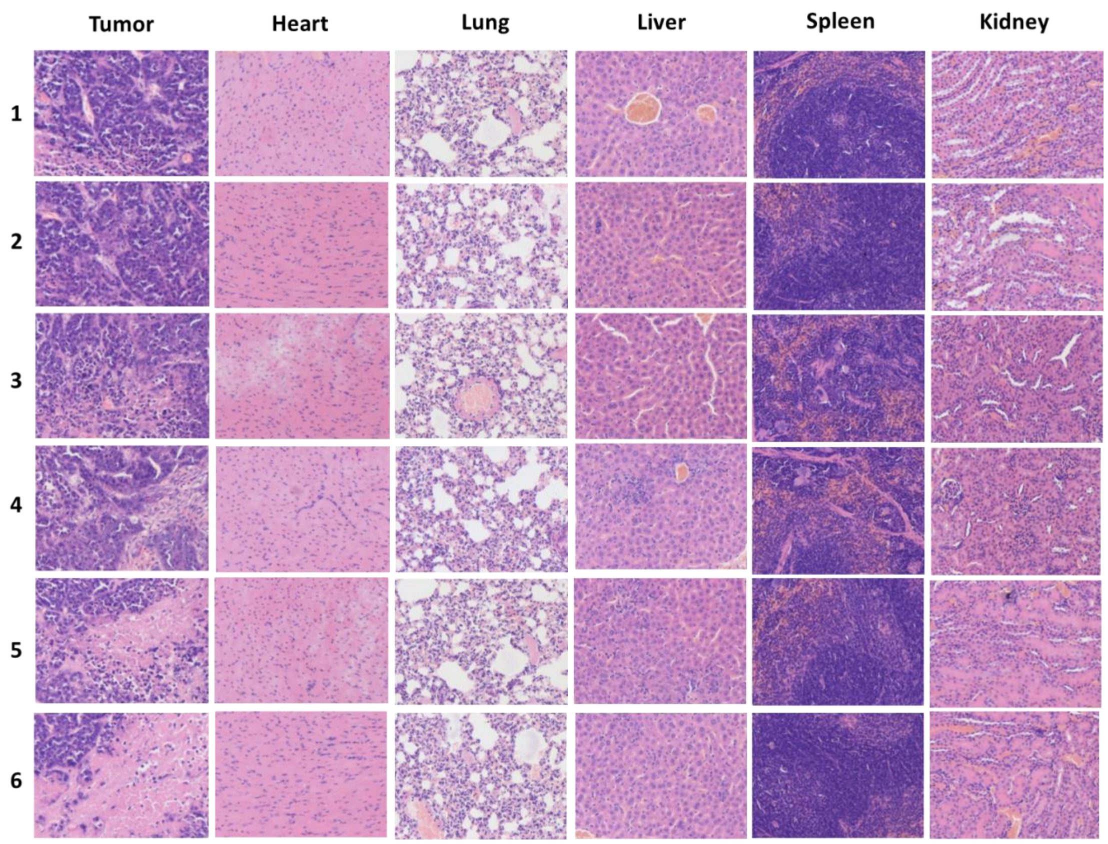

Figure S6. Representative H&E stained images of major organs and tumors after treated with (1) saline, (2) Drug-free NPs@PDA-PEG-Apt, (3) DTX+DOX, (4) DTX/NPs@PDA/DOX-PEG, (5) DTX/NPs@PDA/DOX-PEG-Apt and (6) DTX/NPs@PDA/DOX-PEG-Apt+NIR over 14 days.
